# Sex Differences in Body Image Perception and Ideals: Analysis of Possible Determinants

**DOI:** 10.3390/ijerph19052745

**Published:** 2022-02-26

**Authors:** Emanuela Gualdi-Russo, Natascia Rinaldo, Sabrina Masotti, Barbara Bramanti, Luciana Zaccagni

**Affiliations:** 1 Department of Neuroscience and Rehabilitation, Faculty of Medicine, Pharmacy and Prevention, University of Ferrara, 44121 Ferrara, Italy; emanuela.gualdi@unife.it (E.G.-R.); luciana.zaccagni@unife.it (L.Z.); 2 Center for Exercise Science and Sports, University of Ferrara, 44121 Ferrara, Italy; 3 Department of Environmental and Prevention Sciences, Faculty of Medicine, Pharmacy and Prevention, University of Ferrara, 44121 Ferrara, Italy; barbara.bramanti@unife.it; 4 Center for Studies on Gender Medicine GMC-Unife, University of Ferrara, 44121 Ferrara, Italy

**Keywords:** body image perception, dissatisfaction, misjudgment, sex, body composition, exercise

## Abstract

The study analyzed the differences between sexes in body image perception and body ideals to assess possible dissatisfaction and misinterpretation in the body image considered attractive for the other sex. Moreover, the influence of anthropometric traits and sports practice on body dissatisfaction and misjudgment was evaluated. Using a cross-sectional design, 960 Italian university students were investigated. Anthropometric characteristics were measured directly. Assessment of body image perception was performed using Thompson and Gray’s silhouettes. We developed two new indexes to assess the possible discrepancy between (1) the perceived silhouette of one’s body and that of the same sex deemed attractive to the other sex (FAD); (2) the silhouette is deemed attractive to the opposite sex and the average attractive silhouette selected by the opposite sex (AMOAD). As expected, females showed greater dissatisfaction with their bodies than males concerning both their own ideal and the silhouette they considered attractive to the opposite sex. Although both sexes misjudged the attractive silhouette for the opposite sex, women were found to be more wrong. According to the outcomes of multivariate regression models, stature, body composition parameters, amount of sport, sex, and FAD were significant predictors of dissatisfaction and misjudgment. In addition to action aimed at correcting misperceptions, the study revealed the importance of sports participation in improving the perception and acceptance of one’s body image.

## 1. Introduction

Body image is a complex, multidimensional and dynamic concept that encompasses a person’s perception, thoughts, and feelings about his/her body [1]. Body image perception has been found to be influenced by several factors, such as socio-demographic factors, physical activity (PA), stress, and nutritional behaviors and it is connected with nutritional and mental disorders [2,3,4]. It is well known that body image disturbance, which includes body image dissatisfaction and body image misperception, is related to eating disorders, abnormal eating habits, and unhealthy weight control behaviors [5,6]. In particular, body image dissatisfaction has been connected to several negative health outcomes, including frequent dieting, bulimia, dietary restraint [7,8], and the development of related psychopathologies, such as depression [9]. Previous research has analyzed the extent of body image dissatisfaction according to sex and gender [10,11], life span [12,13,14], and culture [15,16,17]. It is well known that a greater amount of body dissatisfaction is found in women in comparison to men, with a peak especially during young adulthood [18]. The other face of body image is body image distortion, which is the misperception of one’s own real body. It is considered a growing health concern as it is affecting both women and, increasingly, men [2,4,19,20,21,22].

However, most studies have just analyzed body dissatisfaction between what persons think they are and what they want to be. Up to date, just a few and non-recent studies have analyzed the relationship between the differences in perceived attractiveness between the two sexes [23,24,25,26,27,28], but none of them proposed a quantitative method to measure the dissatisfaction arising between what a person thinks he/she is and what he/she thinks that the other sex finds attractive. Indeed, especially in young adulthood, both men and women are oriented towards the search for a permanent mate, thus increasing the concern for what the opposite sex finds attractive and the idealization of slimness, especially in young women [29,30]. Studies focusing on attractiveness and misperceptions of what the other sex finds attractive should be implemented.

There are two lines of interpretation when talking about attractiveness: the evolutionary perspective, focused on how natural selection influences human appearance, valuing those traits that seemed to maximize the chances of reproductive success; the Sexual Strategies Quality hypothesizes that attractiveness reflects physiological healthy and fertile potential mates [31,32]. The other perspective regards a socio-cultural approach, which states that culture has a major role in determining body ideals [33]. According to this theory, the ideal body is based on a social and cultural construct. Several studies have found that a low Waist to Hip Ratio (WHR) (about 0.70) and a Body Mass Index (BMI) of approximately 18–19 kg/m^2^ is considered the most attractive in female bodies in Western societies [34,35], whereas the most attractive male body for women is characterized by high muscle mass, higher BMI (about 26 kg/m^2^) and low Waist to Chest ratio (about 0.70) [30]. Another study demonstrated that the most attractive amount of body fat in women’s bodies is slightly below the healthy range [36]. All of these findings underlined the idealization of thinness, especially among females, and, as a consequence, the increased risk of body dissatisfaction and the low self-esteem that leads to body image misperception and distortion [37,38].

Studies focusing on the misperception of attractiveness have demonstrated that women generally have an inaccurate perception of what males find attractive. In detail, it has been shown that females tend to underestimate the males’ ideal female body, thus decreasing the satisfaction with their own bodies. This phenomenon was first studied in 1985 by Fallon and Rozy [23] and was then confirmed by other studies [24,25,26,27,28]. Moreover, Brewis and co-workers demonstrated that women misperceived also what other women consider the most attractive body [39]. In contrast, men are more aware of the ideal male body for females [24,25,26,27,28]. The study of Bergstrom et al. [28] found an association between low self-esteem, misperception of other sex ideal body and eating disorders. The need for up-to-date studies is underlined by the fact that attractiveness and contingent self-esteem vary across the lifespan and generations [25,28,40].

Moreover, no studies examined the relationship between attractive body image and physical activity (PA), and/or sports practice. It is well known from the literature that the practice of PA decreases the level of dissatisfaction and misperception of one’s own body in both children and adolescents [41,42], as well as in adults [11,21,43]. However, it is not known whether the practice of PA might increase awareness of which body the other sex finds most attractive and the influence between the ideal of the other sex and the ideal of one’s person.

The aims of this study are: (i) to analyze young women’s and men’s perceptions of attractive body images for themselves and peers of the opposite sex and the differences between the sexes; (ii) to propose a method to evaluate the level of dissatisfaction between the perception of one’s own body and the silhouette perceived as attractive to the opposite sex, and a method for assessing the misjudgment between sexes in defining the silhouette deemed ideal by the opposite sex; (iii) to define the anthropometric and PA variables associated and possible predictors of dissatisfaction and misperception.

## 2. Materials and Methods

### 2.1. Participants and Procedures

A cross-sectional study was carried out on a sample of 960 Italian university students (378 females aged 21.2 ± 1.9 years, and 582 males aged 21.6 ± 2.6 years) from the Sports Science degree program (Faculty of Medicine and Prevention, University of Ferrara, Italy) (survey years: 2012–2019). The recruitment of students as research participants was done on a voluntary basis, advertised through general announcements, and ensured the anonymity of the data collected. Students interested in participating in the study, responding to the offer of a free body composition assessment, received detailed information about the research and requirements from the researchers. The following inclusion criteria were required: (i) being in the age range 19–30 years; (ii) being Italian; (iii) having no diagnosed health problems that may interfere with anthropometric measurements or body image perception; iv) both anthropometric and body image perception assessments have been completed including personal data and self-reported information about sports practice (yes/no and amount in hours/week). All volunteers gave written informed consent, and the research was approved by the Ethic Committee for Biomedical Research of the University of Ferrara.

### 2.2. Anthropometric Measurements

Anthropometric characteristics were measured by researchers and trained staff according to standardized procedures [44,45]. Weight was measured using a mechanical scale (precision 0.1 kg; SECA GmbH & Co. KG., Hamburg, Germany) and stature was determined with a wall-mounted stadiometer (precision 0.1 cm; Magnimeter, Raven Equipment Limited, Dunmow, Essex, UK).

Body Mass Index (BMI) was calculated using the formula weight/stature^2^ (kg/m^2^) to assess the weight status of the participants according to the World Health Organization (WHO) cut-points [46]. Students were categorized as underweight, normal weight, overweight, and obese (actual weight status encoded as 1, 2, 3, and 4, respectively).

Waist circumference (WC) and skinfold thicknesses at triceps (T Sk) were measured to the nearest 0.1 cm using a non-stretchable tape (DKSH, Zurich, Switzerland) and a Lange caliper (Beta Technology Inc., Cambridge, MD, USA), respectively. In addition, we classified participants at risk for central obesity by WC using cut-points ≥88 cm for females and ≥102 cm for males, according to guidelines from the National Institute for Health and Clinical Excellence [47].

Body composition parameters (percentage of Fat mass (%F), Fat Mass (FM, kg), and Fat-Free Mass (FFM, kg) were calculated using the skinfold equations developed by Durnin and Womersley [48] and Siri [49]. Subjects were classified as under fat, normal fat, over fat, and very overfat based on sex and age cut-points of fat mass percentage according to Gallagher et al. [50].

### 2.3. Body Image Assessment

The body image perception was assessed according to a silhouette-matching technique. The scale of silhouettes used in this study was developed by Thompson and Gray [51] and has been modified by removing facial features from the silhouettes because they might influence the choice [16]. The scale consists of nine silhouette drawings for each sex numbered in increasing order from the slimmest (silhouette 1) to the largest (silhouette 9). The silhouette series were divided according to the BMI categories determined by Cole’s [52] cut-off values (1 = underweight status, 2 = normal weight status, 3 = overweight status, and 4 = obese status). Silhouettes 1 and 2 correspond to underweight, silhouettes 3, 4, and 5 correspond to normal weight, silhouettes 6 and 7 correspond to overweight, silhouettes 8 and 9 correspond to obesity. Participants were asked to choose the silhouette best representing how they currently look (feel), the silhouette showing how they ideally wanted to look (ideal), the silhouette they thought the opposite sex found most attractive (attractive) and the silhouette they found most attractive among the silhouettes of the opposite sex (other attractive). Participants could not choose an intermediate value between those of two successive silhouettes.

FID (Feel minus Ideal Discrepancy) index was used to assess the degree of body dissatisfaction, as feel silhouette minus ideal silhouette [53]. A positive score indicates the desire to be thinner and a negative score indicates the desire to be fatter. An FID score of 0 indicates no dissatisfaction (same silhouette chosen as feel and as ideal).

FAI (Feel weight status minus Actual weight status Inconsistency) index was used to measure the improper perception of own actual weight status subtracting it from the weight status of the feel silhouette according to a precise encoding [21]. Positive FAI scores indicate that weight status is overestimated, while negative ones indicate that weight status is underestimated. Realistic perception of one’s weight status is designated by the score 0.

We developed a new index, named FAD (Feel minus Attractive Discrepancy), to evaluate the discrepancy between the feel silhouette and the silhouette of same-sex perceived attractive for the opposite sex (attractive silhouette). A FAD score of 0 indicates a correspondence between the feel silhouette and the attractive silhouette for the opposite sex. When the score is different from 0, the discrepancy amount reflects the degree of dissatisfaction. A positive FAD score indicates that the feel silhouette is fatter than the attractive silhouette for the opposite sex; a negative FAD score indicates that the feel silhouette is leaner than the attractive silhouette for the opposite sex.

Another index, named AMOAD (Attractive minus Mean of Other Attractive Discrepancy), was developed to assess the eventual misjudgment between the silhouette perceived as attractive to the opposite sex and the average attractive silhouette chosen by the opposite sex. An AMOAD score of 0 indicates a correspondence between the attractive silhouette and the mean of other attractive silhouettes. A positive AMOAD score indicates an overestimation of the silhouette chosen as attractive to the opposite sex and a negative AMOAD score indicates an underestimation of the same.

Table 1 provides a summary of the various silhouettes and indices used to assess body image perception.

### 2.4. Statistical Analysis

Descriptive statistics were calculated for anthropometric traits, weight- and fat-status, and body image perception data and were expressed as mean values and standard deviation (SD), or frequencies, separately by sex.

Distribution normality was assessed using Kolmogorov–Smirnov test. For statistical comparisons, the log-transformed values of skinfolds at triceps were computed to normalize the distribution, as is usually done [21,48,54].

Differences between sexes in the categorical variables were tested by the chi-square test and differences in the means of the continuous variables by the Student’s *t*-test or Mann–Whitney test when the examined traits were found to have a normal or non-normal distribution, respectively.

Backward multiple regression analysis was used to assess possible predictors of FID, FAD, and AMOAD. Anthropometric traits, sex (male/female), sports practice (yes/no), and weekly amount of sport were included in the regression models as independent variables. Sex was included in the model as a binary variable with males as the reference group (male coded 0 and female coded 1). Predictor inputs with significant association were inserted into the model (*p* < 0.05), while those non-significant were removed from the model. Multicollinearity among the variables is examined by variance inflation factors (VIFs), assuming VIF values between 0.10 and 10 as acceptable [55]. Values of *p* < 0.05 were assumed to be statistically significant.

All the analyses were carried out with “Statistica” for Windows, Version 11.0 (StatSoft Srl, Tulsa, OK, USA).

## 3. Results

Table 2 shows anthropometric characteristics of the sample by sex. As expected, on average males were significantly taller and heavier, with a higher BMI, a larger WC, and a thinner T Sk than females. Concerning body composition parameters, males had less fat mass (both absolute and relative), and more fat-free mass than females.

Males were sportier than females (88.7% vs. 84.1%) and played significantly more sport per week (6.8 h vs. 5.5 h).

Table 3 shows the distribution of the sample according to weight status, fat status, and WC. Three-quarters of the sample were normal weight and females significantly (*p* < 0.01) differed from males: women were five times more likely than men to be underweight, and half as likely as men to be overweight and obese. A similar trend was observed in fat-status (*p* < 0.01): under-fat and normal fat categories were more represented in women, while overfat and very overfat categories were more represented in males. As regards WC, less than 2% of the subjects were above the cut-off (≥88 cm for women and ≥102 cm for men) at risk for centralized obesity in both sexes.

Table 4 shows the analysis of body image perception by sex. Women chose on average a feel silhouette (4.96) in the range of normal weight and normal fat and a significantly *(p* < 0.001) thinner silhouette as ideal (3.84), so their dissatisfaction (expressed using the FID index) was positive and slightly above 1. The silhouettes chosen by males were, on average, significantly bigger than those chosen by females both in terms of feel and ideal silhouettes, but the mean value of the ideal silhouette (5.18) was not significantly smaller (*p* = 0.073) than the feel one (5.28). Females yielded a significantly higher FID value than males, which demonstrated a higher dissatisfaction in young women due to their wish to be slimmer than they actually were. In particular, in the female sex, only 16.9% had FID = 0, i.e., the feel silhouette corresponds to the ideal silhouette; 5.9% had FID < 0, i.e., they wanted to be heavier, and 76.8% had FID > 0, i.e., they wanted to be slenderer; in the male sex, the three groups were more homogeneous, with 30.2% of subjects with FID = 0, 30.8% with FID < 0, and 37.6% with FID > 0.

Women had on average a positive FAD index and significantly higher than men: the feel silhouette was 1.02 bigger than the attractive silhouette while men’s FAD value was only 0.34, indicating a lower dissatisfaction than women. Women selected an attractive silhouette slimmer than their feel but a little bigger than ideal; men selected a slimmer attractive silhouette than their feel and an even slimmer silhouette as ideal. In particular, in the female sex, only 14.6% had FAD = 0 (that is the feel silhouette corresponds to the attractive silhouette), 15.9% had FAD < 0 and 65.2% had FAD > 0; in the male sex 20.5% had FAD = 0, 29.4% had FAD < 0 and one-half had FAD > 0.

Both sexes demonstrated a good perception of their body, as revealed by the FAI mean values close to 0 and slightly positive, indicating a little tendency to overestimate one’s weight status and a similar trend in men and women (*p* = 0.073).

In our sample of young adults, both sexes misperceived what the opposite sex finds attractive. Women overestimated the extent to which men prefer thin women: on average, women thought that men preferred women to be thinner (3.93 ± 0.93) than men actually reported finding attractive (4.53 ± 0.81) and the difference resulted highly significant (*p* < 0.001). Additionally, in the male sex, men thought that women preferred men to be thinner (4.94 ± 0.75) than women actually reported finding attractive (5.08 ± 0.57) and the difference was significant (*p* = 0.002). The difference between the attractive silhouette and the mean value of the actual attractive body silhouette reported by the other sex confirmed that men were more accurate than women in predicting the body size found most attractive by their opposite-sex peers (*p* < 0.001).

**Figure 1:**
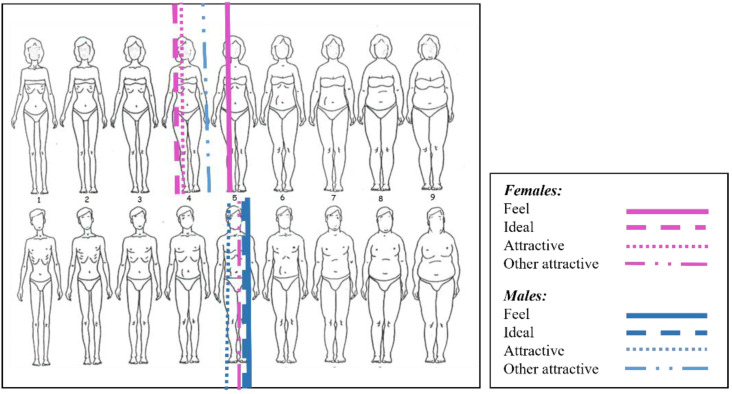
Representation of body image perception in both sexes using the body silhouette chart by Thompson and Gray (1995) (reproduced for the kind permission of John Wiley & Sons, Inc., Hoboken, NJ, USA).

Using the silhouette scheme proposed by Thompson and Gray [51], we reported the mean values of the different silhouettes, thus highlighting the greater discrepancy between the silhouette perceived as real (feel) and ideal silhouettes (ideal and attractive) found in the female sex (Figure 1).

Table 5 shows the multiple linear regression analysis for the whole sample, conducted with all the variables input into the model with a backward method. The analysis was used to investigate whether the FID of the subjects could be explained by their characteristics, namely sex, sports practice (yes/no), amount of sport (hours/week), and all the anthropometric characteristics. The model showed a significant association of FID with WC, %F, and FFM (positive), and with stature and to be a male (negative). These independent variables accounted for 46.2% of the variance of FID in young adults.

Table 6 shows the multiple linear regression analysis for the FAD as the dependent variable. In the multivariate model being a male, stature, and weekly amount of sports practice were significant predictors negatively associated with FAD, while WC, FM, and FFM were positively associated with it. The model accounted for 41.0% of the variance of FAD.

Table 7 shows the model of multiple linear regression analysis for the dependent variable AMOAD in the whole sample. Among all the variables (included FAD) input into the model with a backward method, the stature, BMI, and WC were positively associated with AMOAD, and FFM, FAD, and weekly amount of sports practice were negatively associated. The model accounted for 45.7% of the variance.

## 4. Discussion

This study showed that the perception of one’s body silhouette differs significantly from that believed to be attractive to the opposite sex. Moreover, the perception of the body silhouette that the opposite sex believes is attractive is different from what the opposite sex actually considers attractive. Dissatisfaction both for one’s ideal and for the silhouette considered attractive to the opposite sex was found to be more widespread in females than in males, indicating that ideal and attractive silhouettes were thinner than the silhouette perceived as actual.

Regarding the anthropometric characteristics of the sample examined, there was a percentage of 18.5 of overweight/obese individuals, which is lower than the national percentage: four Italian adults out of 10 (age range 18–69 years) are overweight/obese according to the Istituto Superiore di Sanità (Sorveglianza PASSI) [56]. Clearly, the younger age of our sample and the possible effectiveness of university educational interventions targeting healthier lifestyles may have influenced this result. However, looking at the sexes separately, the percentage of overweight/obese men was about twice the percentage of women in our sample. The female subsample, besides having a higher percentage of normal weight, also showed a frequency of underweight that was five times higher than in the male subsample. In terms of fat status, women show a percentage of under fat that is twice as high and of overfat that is one-fifth compared to the male sex. In both sexes, there is a net prevalence of normal fat and normal weight. Females had a higher prevalence of central obesity than males, as is generally found in all populations [57]. The perception of one’s own body revealed by the choice of the feel silhouette with respect to the relative anthropometric data expressed by the FAI was appropriate in both sexes, although both showed a tendency to overestimate their weight status.

The ideal male and female body silhouettes were thinner than the perceived real body silhouette, although dissatisfaction was stronger for women with significant differences between real and ideal silhouettes. Consequently, dissatisfaction with one’s body image (FID) was only found in this sex, confirming that physical appearance is considered more important by females than by males in agreement with the results of previous studies [11,21,58,59]. Although higher frequencies of overweight/obese were observed among males, this study confirmed that they are less aware of their weight status than women, as already reported in the literature [60,61,62].

The silhouette considered attractive to the opposite sex differs little from the ideal silhouette with a slight tendency towards a slightly thinner silhouette in men and a slightly less thin in women. We developed and applied a new index (FAD) to evaluate any discrepancy between the silhouette perceived as real and the one deemed attractive to the opposite sex. Again, males were more satisfied than females with their body image as confirmed by FAD values that differed little from the 0 value (indicating an almost complete overlap of the actual silhouette with the attractive one).

We calculated the individual misjudgment (AMOAD) by subtracting the mean value of the attractive silhouette reported by the opposite sex from the individual’s own estimate of the silhouette preferred by the opposite sex. It was thus apparent that, although both sexes believed that the silhouette attractive to the opposite sex must be thinner than their feel silhouette, the misjudgment was more pronounced in women who believed that there was a preference of men for very thin women. Our results contrast with findings on populations of no industrialized countries and at low socioeconomic levels [37,39,63], whereas they are consistent with those observed on populations of industrialized countries or at high socio-economic levels [25,31,37]. The ideal of thinness, launched by the fashion industry since the 1960s, has become and still remains the idealized standard of beauty typical of Western cultural values [64], exerting its influence especially on the female sex.

Dissatisfaction can have serious consequences on people’s lifestyle behaviors and eating habits. It represents therefore a major problem since dissatisfaction with one’s body image is widespread and seems to affect three-quarters of the Mediterranean adult populations [11]. Given the importance of this aspect, we attempted to identify the determinants of dissatisfaction with perceived real body image versus ideal body image (FID) or to body image deemed attractive to the opposite sex (FAD). Among the variables analyzed as dependent in the multivariate regression analysis, stature, WC, %fat, FFM, and sex were found to be significant determinants of FID. In particular, FID (i.e., dissatisfaction) decreased with increasing stature and in the male sex compared to the female, while it was found to increase with increasing WC, %F, and FFM. This last result should not be surprising, as the body silhouettes adopted in this research to assess eventual dissatisfaction may only depict variations in weight status, that is affected by both FM and FFM. Other studies based on different evaluation methods have focused on other aspects, such as muscularity, which may be a concern for many men [65].

Concerning the other determinants, as we have already pointed out, sex plays a decisive role with women desiring increased thinness following the standard of beauty promoted by mass media in Western countries. In addition, this desire is modulated by the belief that a thin body is more attractive to men and that men are more interested in women who are thinner than average [63].

Interestingly, as stature increases, dissatisfaction decreases. After all, stature contributes to the overall body shape and is part of the weight index (BMI) on which silhouettes are based. The feminine ideal standard of beauty is not only thin but also tall [11,66]. In short, this ideal is characterized by a strong tendency toward an ectomorph body type.

Among the determinants of dissatisfaction with the perceived body image compared to what is assumed to be attractive to the opposite sex (FAD), stature and sex emerge again, with a trend similar to that already shown for FID. In addition, among the anthropometric variables, FM, FFM, and WC are also found to significantly influence FAD, which is found to increase as they increase. Finally, the number of hours spent in sport activities emerged as an additional predictor with an opposite influence on FAD: dissatisfaction decreases as the number of hours of sport activity increases. This result is consistent with the findings of Ouyang et al. [67], according to which body image is significantly related to sport participation, and body image increases with sport participation in college students. These results are also in agreement with the review published by Hausenblas and Downs, reporting that athletes had a more positive body image compared to non-athletes [68]. However, the reason for this evidence is still debated. Indeed, it is unclear whether positive body image is a consequence or a cause of sport participation, i.e., whether it is the practice of sport that increases body image satisfaction and self-esteem or whether athletes have a more “ideal” physique due to their athletic endeavors.

The limitations of this study depend primarily on the fact that the research design is cross-sectional. Future research may possibly validate our findings through a longitudinal study. Furthermore, this study analyzed a homogeneous sample in terms of age (19–30 years) and ethnicity (Italian). Since attractiveness standards are thought to differ by ethnicity and by age according to some studies [15,63,69], while the results of the study by Cachelin et al. [70] did not support ethnic differences in body image (controlling for age, education level, and BMI factors), further research is needed to test the validity of our findings in other populations and age groups. In addition, the study lacks assessment of eating disorders, so we do not know how many of our participants may suffer from disordered eating or eating disorders, which might impact our scores. Finally, this study did not consider possible differences in body image perception among students engaged in different sports. However, individual or team sports do not significantly affect the perception of body image according to the literature [68,71]. Future research should also consider the competitive level of the athletes, as elite athletes appear to be more prone to eating disorders and body image dissatisfaction [72], although findings are still mixed in the literature and may depend on the type of sport practiced [73].

## 5. Conclusions

This study showed that dissatisfaction with body image, both with respect to ideal beauty and to the silhouette considered attractive to the opposite sex, is greater in women and increases with increasing body mass (%F and FFM or weight and WC) and decreasing stature. Sports activity also emerged as a determinant factor in the perception of the silhouette considered attractive to the opposite sex. This evaluation was made possible by the new proposed FAD index based on the difference between the silhouette perceived as own real body image and the silhouette of the same sex considered attractive to the opposite sex.

The other index we proposed in this study (AMOAD) made it possible to assess the misperception of body image considered attractive to the opposite sex compared with that claimed by the opposite sex. In this respect, both men and women misjudged the silhouette considered attractive by the opposite sex, but the female sex failed more than the male sex. Since both dissatisfaction and misjudgment may be associated with more negative eating and body attitudes and disordered eating behaviors [32], it is important to take these perceptions into account by seeking, when possible, to correct them to prevent the onset of eating disorders. Social and individual educational interventions aimed at avoiding the promotion of unrealistic body images, correcting misperceptions, and promoting sports participation can be useful strategies to improve the perception and acceptance of own body image and to increase self-esteem.

## Figures and Tables

**Table 1:** List of silhouettes and indices for the assessment of body image perception.

Silhouette	Significance
Feel	Silhouette that best represents how a person currently looks
Ideal	Silhouette that best represents how a person ideally wants to look
Attractive	Silhouette that a person thinks the opposite sex finds most attractive
Other attractive	Silhouette that a person finds most attractive among the silhouettes of the opposite sex
**Index**	
FID	Feel minus Ideal Discrepancy
FAI	Feel weight status minus Actual weight status Inconsistency
FAD	Feel minus Attractive Discrepancy
AMOAD	Attractive minus Mean of Other Attractive Discrepancy

**Table 2:** Comparison by sex in anthropometric characteristics and sports amount.

	Females *n* = 378			Males *n* = 582			
**Variables**	**Mean**	**SD**	**Range**	**Mean**	**SD**	**Range**	***p***
Stature (cm)	163.9	6.5	144.4–186.5	177.6	7.1	147.5–199.4	<0.001
Weight (kg)	59.2	8.8	36.0–91.7	74.3	10.5	43.4–130.0	<0.001
BMI (kg/m^2^)	22.0	2.8	16.7–35.7	23.5	2.8	17.2–37.5	<0.001
WC (cm)	70.3	5.9	58.1–95.2	79.9	6.9	57.9–95.1	<0.001
T Sk (mm)	18.7	5.5	7–38	11.6	5.5	3–41	<0.001
%F	28.5	4.6	14.2–40.4	17.4	4.7	5.0–31.7	<0.001
FM (kg)	17.0	4.6	6.8–33.2	13.1	4.5	3.1–28.8	<0.001
FFM (kg)	42.2	5.4	25.1–60.2	61.2	8.3	33.2–101.7	<0.001
Sports amount (h/week)	5.5	3.8	0–20	6.8	3.9	0–21	<0.001

WC = waist circumference; T Sk = skinfold thickness at triceps; %F = fat percentage; FM = fat mass; FFM = fat free mass.

**Table 3:** Distribution of subjects according to weight status, fat-status, and waist circumference.

	Females	Males	*p*
**BMI**	***n* (%)**	***n* (%)**	<0.01
Underweight	32 (8.6%)	9 (1.6%)	
Normal weight	293 (78.8%)	426 (74.1%)	
Overweight	40 (10.8%)	122 (21.2%)	
obese	7 (1.9%)	18 (3.1%)	
**%F**			<0.01
Under fat	27 (7.1%)	22 (3.8%)	
Normal fat	286 (75.7%)	402 (69.1%)	
Over fat	61 (16.1%)	126 (21.6%)	
Very overfat	4 (1.1%)	32 (5.5%)	
**WC**			0.59
Females ≥ 88 cm	7 (1.9%)	-	
Males ≥ 102 cm	-	7 (1.2%)	

BMI = body mass index; %F = fat percentage; WC = waist circumference.

**Table 4:** Comparison of body image perception by sex.

	Females *n* = 378		Males *n* = 582		
Body Image Perception	Mean	SD	Mean	SD	*p*
Feel silhouette	4.96	1.31	5.28	1.13	0.001
Ideal silhouette	3.84	0.93	5.18	0.59	<0.001
Attractive silhouette	3.93	0.93	4.94	0.75	<0.001
Other attractive silhouette	5.08	0.57	4.53	0.81	<0.001
FID	1.13	1.06	0.09	1.11	<0.001
FAD	1.02	1.45	0.34	1.34	<0.001
FAI	0.26	0.56	0.19	0.54	0.151
AMOAD	−0.60	0.93	−0.14	0.75	<0.001

FID = feel minus ideal discrepancy; FAD = feel minus attractive discrepancy; FAI = feel weight status minus actual weight status inconsistency; AMOAD = attractive minus mean of other attractive in opposite sex discrepancy.

**Table 5:** Predictors of FID: results of multiple linear regression with the backward method.

Predictor	β	t	*p*	VIF
Stature	−0.462	−10.110	<0.001	3.149
WC	0.164	3.009	0.003	4.498
%F	0.437	8.836	<0.001	3.693
FFM	0.653	8.497	<0.001	8.918
Sex (male)	−0.392	−7.260	<0.001	4.389
*R^2^*	0.465			
*R^2^ adjusted*	0.462			
*p*	<0.001			

β: standardized regression coefficient; VIF = variance inflation factor; WC = waist circumference; %F = fat percentage; FFM = fat free mass.

**Table 6:** Predictors of FAD: results of multiple linear regression with the backward method.

Predictor	β	t	*p*	VIF
Stature	−0.475	−9.89	<0.001	3.185
WC	0.169	2.940	0.003	4.587
FM	0.342	8.609	<0.001	2.187
FFM	0.610	8.356	<0.001	7.370
Sex (male)	−0.352	−6.537	<0.001	4.010
Amount of sports	−0.067	−2.411	0.016	1.073
*R^2^*	0.414			
*R^2^ adjusted*	0410			
*p*	<0.001			

β: standardized regression coefficient; VIF = variance inflation factor; WC = waist circumference; FM = fat mass; FFM = fat free mass.

**Table 7:** Predictors of AMOAD: results of multiple linear regression with the backward method.

Predictor	β	t	*p*	VIF
Stature	0.208	3.385	0.001	5.704
BMI	0.473	8.188	<0.001	5.022
WC	0.195	3.732	<0.001	4.098
FFM	−0.337	−4.378	<0.001	8.916
FAD	−0.783	−24.194	<0.001	1.576
Amount of sports	−0.059	−2.193	0.029	1.075
*R^2^*	0.461			
*R^2^ adjusted*	0.457			
*p*	<0.001			

β: standardized regression coefficient; VIF = variance inflation factor; BMI = body mass index; WC = waist circumference; FFM = fat free mass; FAD = feel minus attractive discrepancy.

## Data Availability

Not applicable.
